# Immune dysregulation in Glycogen Storage Disease 1b - a CyTOF approach

**DOI:** 10.21203/rs.3.rs-2598829/v1

**Published:** 2023-02-23

**Authors:** Arne Gehlhaar, Dror Shouval, Eduardo Gonzalez Santiago, Galina Ling, Blake McCourt, Lael Werner, Baruch Yerushalmi, Liza Konnikova

**Affiliations:** Yale University School of Medicine

**Keywords:** GSD1b, neutropenia, immunodeficiency, CyTOF, immunity, immunometabolism

## Abstract

Glycogen Storage Disease type 1b (GSD1b) is a rare disease manifesting as hypoglycemia, recurrent infections and neutropenia, resulting from deleterious mutations in the *SLC37A4* gene encoding the glucose-6-phosphate transporter. The susceptibility to infections is thought to be attributed not only to the neutrophil defect, though extensive immunophenotyping characterization is currently missing. Here we apply a systems immunology approach utilizing Cytometry by Time Of Flight (CyTOF) to map the peripheral immune landscape of 6 GSD1b patients. When compared to control subjects, those with GSD1b had a significant reduction in anti-inflammatory macrophages, CD16^+^ macrophages, and Natural Killer cells. Additionally, there was a preference towards a central versus an effector memory phenotype in multiple T cell populations, which may suggest that these changes stem from an inability of activated immune cell populations to undergo the appropriate switch to glycolytic metabolism in the hypoglycemic conditions associated with GSD1b. Furthermore, we identified a global reduction of CD123, CD14, CCR4, CD24 and CD11b across several populations and a multi-cluster upregulation of CXCR3, hinting at a potential role of impaired immune cell trafficking in the context of GSD1b. Taken together, our data indicates that that the immune impairment observed in GSD1b patients extends far beyond neutropenia and encompasses innate and adaptive compartments, which may provide novel insights into the pathogenesis of this disorder.

## Introduction

Glycogen Storage Disease type 1 (GSD1), also known as Von Gierke disease, is an autosomal recessive[1] disease, characterized by repeated episodes of hypoglycemia due to inability to break down glycogen storage. The prevalence of GSD1 ranges from 1 in 20,000[2] to 1 in 400,000[3] depending on study and the ethnic background of subjects. GSD1 can be further divided into two categories, GSD1a, resulting from a mutation in the glucose-6-phosphatase catalytic subunit 1 (*G6PC7*)[4], leading to a deficiency of glucose-6-phosphatase catalytic activity, and GSD1b, resulting from mutations in the glucose-6-phosphate (G6P) exchanger *SLC37A4*[4], blocking the transport of G6P into the endoplasmic reticulum. Both mutations lead to an inadequate conversion of G6P into glucose, impacting glycogenolysis and gluconeogenesis pathways, with subsequent hypoglycemia and buildup of glycogen and adipose tissue in liver and kidneys[4]. Clinical manifestations are typically noticeable after 3 to 4 months of age and include hepatomegaly, nephromegaly, hypoglycemic seizures, and failure to thrive[1], [4]. Patients with GSD1 also suffer from short stature with thin extremities, delayed puberty and a protuberant abdomen caused by the massive hepatomegaly[4]. Interestingly, a significant number of patients with GSD1b suffer from inflammatory bowel disease (IBD), typically with a Crohn’s disease phenotype[5], [6].

A unique phenotype in patients with GSD1b is reoccurring infections, that traditionally have been attributed to either associated neutropenia or dysfunctional chemotaxis and intracellular bacterial killing of neutrophils. The cause of neutropenia in GSD1b patients remains unclear. Evidence suggests that the decreased neutrophil abundance and activity is a consequence of increased apoptosis of neutrophils in affected individuals[7]. However, the neutropenia alone cannot solely explain the immunodeficiency state in this disorder, since GCSF therapy doesn’t necessarily eliminate the risk of infections. Therefore, it is likely that the susceptibility to infections is likely attributed to additional effects on other immune subsets. As such, Melis and colleauges[8] were able to observe lymphopenia in patients with GSD1b, but not in GSD1a patients, with a reduction in CD4^+^ T cells, CD8^+^ T cells, and natural killer (NK) cells, compared to controls.

We hypothesized that patients with GSD1b exhibit a broad range of immune dysregulation, beside in the neutrophil compartment, leading to an immunodeficiency phenotype with inflammatory features. In the present study, we utilized Cytometry by Time of Flight (CyTOF) to perform deep immunophenotyping of peripheral blood mononuclear cells (PBMCs) obtained from 6 patients with GSD1b and 3 control subjects to show dysregulation in several immune populations in patients with GSD1b.

## Methods

### Subject Recruitment

The study was approved by the local IRB committees. After informed consent was obtained blood samples were collected from participants.

### Collection and isolation of PBMCs

PBMCs were isolated by density gradient with Lymphoprep^™^ (Stemcell^™^ Technologies) per vendor instructions[9].

### CyTOF Staining

Cells were stained with a cocktail of antibodies per our previously published protocol[10]. In short, cells were stained with Rh103 for viability, washed, blocked with Fc-Block, and incubated with the cocktail of metal-coupled surface antibodies (**Supplementary Table 1**) for 30 minutes at room temperature. Cells were then fixed in 1.6% formaldehyde and stained with Ir-DNA intercalator solution. Cells were resuspended in water containing 1:10 dilution of EQ beads and run on a Helios CyTOF machine (Fluidigm, South San Francisco, Calif) at the Yale School of Medicine CyTOF Core.

### Data analysis and Dimensionality reduction

Raw Flow Cytometry Standard (FCS) file were normalized using the Helios2 software and imported into Cytobank (Cytobank Inc.), followed by manual gating of cells positive for both DNA dyes (191 Ir_DNA1, 193Ir_DNA2) of the Intercalator solution applied after fixing (cf. *3.2)*. From these double positive events signals with a 140Ce intensity > 1×10^3^ were excluded as bead signals. Single viable cells were identified by two consecutive gating steps excluding all events with a relative event length >40 and an intensity > 3×10^1^ for the 103Rh intercalating dye applied before fixation (cf. *3.2*). CD45^+^ events were selected as all events with an intensity for 89Y > 1×10^1^ and exported as a separate FCS file for each GSD1b patient and each control. The obtained files were imported into the R 4.1.1 based shiny plugin cytofkit2[11], [12],. The data loading settings were chosen as follows: Merge Method ceil, Fixed number of cells from each FCS file 50,000, Transformation Method cytofAsinh. Dimensionality reduction was performed following t-Distributed Stochastic Neighbor Embedding (t-SNE) algorithm with a perplexity of 30, with maximal 1000 iterations and 42 was utilized as Seed. Clustering was carried out with Rphenograph with a k value of 30: Cellular Progression was set to NULL. Data was graphed in GraphPadPrism 9.2.0 (GraphPad Software). A two-tailed unpaired t-test was used for the comparisons between groups.

### Mean metal intensity calculations and exclusions

The mean metal intensity (MMI) values of individual cells calculated from the FCS files and output by cytofkit2 were averaged for each marker for each patient, with surface markers showing an expression value below 0.5 MMI in both groups being excluded from further analysis, as they are within the range of background signal. For the remaining expressed surface markers, a fold change was calculated as MMI of GSD1b divided by MMI of controls followed by a two-tailed unpaired t-test to identify markers significantly changed in individual clusters. CD45 and CD326 (EpCAM) were included in the marker analysis to observe any potential batch dependent effects, as expected none of the annotated clusters showed CD326 expression; for all but one cluster no significant change in CD45 expression was observed.

## Results

### Patient cohort

We enrolled 6 patients with GSD1b and 3 controls for this study. Gender, age, inflammatory condition, current medications, absolute neutrophil count (ANC), and c-reactive protein (CRP) levels are provided in Table 1. A deleterious mutation in *SLC37A4* gene was identified in all GSD1b patients (Table 1), and all had an ANC below 1000 cells/ mm^3^, indicative of neutropenia. The average age for the control group was 16.3 years compared to 8.2 years for the GSD1b patients (p = 0.039). Two GSD1b patients also had concurrent IBD, and two patients had signs of bronchiectasis.

### Reduction in total NK cells and macrophage/monocyte populations characterizes GSD1b

**Figure 1:**
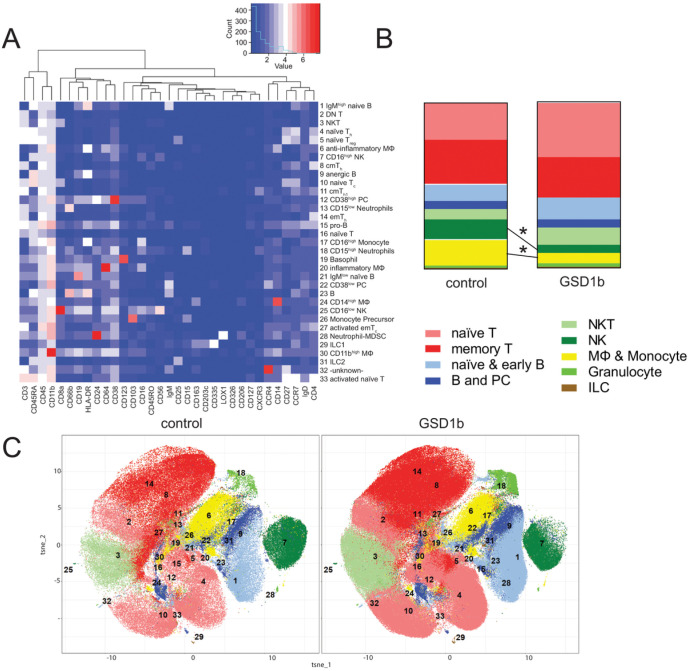
CyTOF clustering shows population differences between GSD1b and control groups. **(A):** Unnormalized expression heatmap output by cytofkit2, markers are shown on the x-, annotated clusters on the y-axis. Abbreviations refer to 5.2) **(B):** Supercluster percentages as part of all CD45^+^ cells, significant reduction was observed in NK cell (p=0.0375) and MΦ & Monoc. (p=0.0049) superclusters. **(C):** t-SNE plots of controls and GSD1b patients, cluster numbers and color legend refer to **(A)-(B).**

To characterize the immune landscape of the peripheral blood of patients with GSD1b vs. controls, we subjected all leukocytes (CD45+ cells) to unsupervised Rphenograph clustering. Automated clustering of the CD45+ populations identified 33 unique populations. The clusters were manually annotated based on average expression values of each marker in each cluster of combined patient samples displayed in the expression heatmap generated by cytofkit2 (Fig. 1A). Utilizing this method, we were able to identify multiple clusters of naive T cells including: CD4^+^ T helper (T_h_) cells, CD25^hi^CD127^lQ^ regulatory T cells (T_reg_), CD4 CD8 double neg (DN) T cells, CD8^+^ cytotoxic T cells (T_c_), a population of activated and non-activated T cells and memory T cells. The B cell compartment was represented by naïve B cells, pro-B cell, activated B cells, plasma cells (PC) and anergic B cells. The innate compartment accessible with our methodology consisted of classical pro- and anti-inflammatory macrophages (MΦ>), CD16^high^ and CD16^low^ monocytes, monocyte precursors, CD15^high^ and CD15^low^ neutrophils, CD16^high^ and CD16^low^ NK cells, a small granulocyte population (Basophil, Neutrophil, Neutrophil – myeloid derived suppressor cells (MDSC)), and innate lymphoid cells (ILC) (Fig. 1A–C). Cluster 32 could not be assigned to a known immune population because of its atypical high expression of CD11c, CD3 and CD19 and likely represented an immune aggregate or an artifact of unsupervised clustering. Given the low overall abundance of this cluster (0.095% in GSD1b cases and 0.13% in controls), it was labeled as unidentified and excluded from further analysis.

To get an overview of the obtained data and determine the differences in the composition of major immune subtypes, the identified clusters were then arranged into superclusters based on their marker similarities (Fig. 1B–C). There was a significant reduction in NK cells (combined clusters 7 and 25; fold change GSD1b/control 0.395, p value = 0.0375) and myeloid cells (MΦ & monocyte cells, combined clusters 6, 17, 20, 24, 26, and 30; fold change GSD1b/control 0.419, p value = 0.0049) in GSD1b patients (Fig. 1B). No significant changes were observed in the remaining superclusters that included naive T cells (clusters 2, 4, 5, 10, 16, and 33), memory T cells (8, 11, 14, and 27), naïve B cells (1, 15, 21, and 32), B cells (9, 12, 22, and 23), NKT cells (3), granulocytes (13, 18, 28, and 19) and ILC (29 and 31) (Fig. 1B).

### NK and myeloid cell percentage changes in GSD1b are population specific

**Figure 2:**
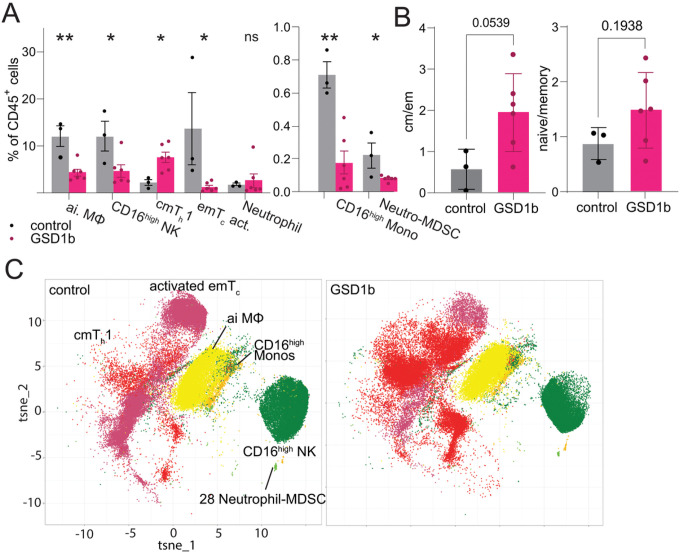
**(A):** significantly changed cluster percentages of clusters 6 p=0.0047; 7 p=0.0379; 11 p=0.0151; 17 p=0.0024; 27 emT_c_ activated p=0.0433; 28 Neutrophil-MDSC p=0.0283. GSD1b patients without IBD nor Bronchiectasis are highlighted in green boxes **(B):** ratios of central memory T cells (clusters 8,11) to effector memory T cells (14,27). **(C):** ratios of naive T cells (2,4,5,10,16,33) to memory T cells (8,11,14,27).

We then determined if there were significant changes at the cluster level. The analysis of innate cell demonstrated a significant reduction in clusters 6, consisting of anti-inflammatory MΦ, and cluster 17, CD16^high^ monocytes (Fig. 2A). The large decrease in anti-inflammatory MΦ observed may explain the overall decrease in the MΦ & monocyte population described above (Fig. 1B). For the NK populations, cluster 7, consisting of CD16^high^ NK cells, was significantly reduced (fold change GSD1b/control = 0.39, p-value = 0.038, resulting in a global decrease in the number of NK cells, Fig. 1B, Fig. 2A, Fig. 2C). The loss of CD16 expression on NK cells is usually associated with their activation[13] and cluster 7 CD16^high^ NK showed significantly lower CD16 levels in GSD1b patients (fold change GSD1b/control = 0.496, p-value = 0.026), indicative of increased activation of NK cells. However, no relevant or significant increase was observed in CD16^low^ NK clusters. While the overall neutrophil populations were similar between patients and controls, cluster 28, Neutrophil-MDSC, was significantly reduced in GSD1b patients (fold change GSD1b/control = 0.359, p-value = 0.0283, Fig. 2B). Nevertheless, assessment of the neutrophilic compartment after a gradient preparation is of limited value and may not provide an accurate estimation of cell composition.

### GSD1b patients exhibit high central memory and low effector memory T cells levels

On the adaptive immunity side, two T cell clusters were altered between GSD1b subjects and controls. Cluster 11, representing central memory T helper 1 cells (cmT_h_1), was increased (fold change GSD1b/control = 3.54, p-value = 0.015, Fig. 2A), while cluster 27, effector memory cytotoxic T cells (emT_c_), was reduced in GSD1b patients (fold change GSD1b/control = 0.09, p = 0.043, Fig. 2A), potentially indicating a dysregulated switch from effector memory to central memory T cells. Moreover, the ratios of central memory T cells (clusters 8 and 11, annotation based on: CD45RA^−^ CD45RO^+^ CCR7^+^ expression) to effector memory T cells (clusters 14 and 27; annotation based on: CD45RA^−^ CD45RO^+^ CCR7^−^expression)[14] in healthy controls and GSD1b was 3.4 times higher in patients with GSD1b, with a confidence interval of 94.61% (p = 0.054, Fig. 2B). Additionally, we observed a trend in the overall reduction of memory T cells and an increase in naive T cells in the GGD1b subjects with an increase in the ratio of naive to memory T cells in GSD1b subjects (Fig. 2A, Fig. 2B).

### Global downregulation of myeloid markers and upregulation of CXCR3 in GSD1b

**Figure 3:**
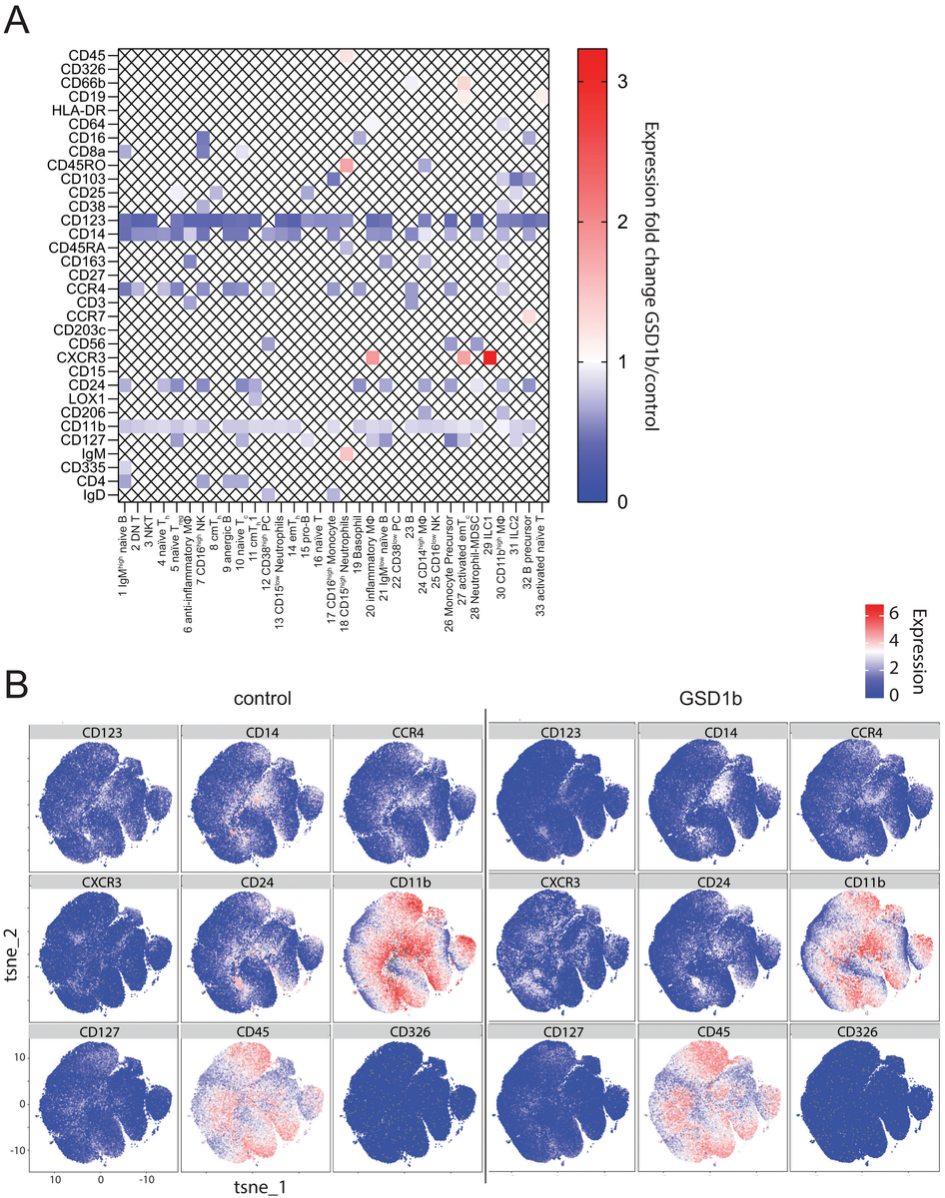
MMI data identifies differential expression of distinct cellular markers across cell populations. **(A):** Heatmap of significant MMI fold changes plotted for each marker and for each cluster, markers with a MMI lower than 0.5 for both clusters and markers with a p>0.05 for a two-tailed unpaired t-test were crossed out. **(B):** Marker Expression Level Plots generated by cytofkit2 depicting ubiquitously changed surface markers in addition to CD45 and CD326.

To assess differential expression of surface markers between the groups, we evaluated the MMI in all the clusters and plotted changes found significant in Fig. 3A (**Supplementary Figure S1B** shows all marker fold changes). Although every cluster demonstrated some differences in marker expression between the two groups and almost all markers were altered in at least some of the clusters, we identified a few markers that were consistently downregulated in a majority of clusters among patients with GSD1b, including CD123 (IL-3Rα), CD14 (receptor for lipopolysaccharide-lipopolysaccharide [LPS] binding protein complex), CCR4 (receptor for CCL17 and CCL22), CD24 (ligand of P-selectin, SELP), CD11b (myeloid lineage marker, subunit of integrin CR3) and CD127 (IL-7R). Interestingly, CXCR3 (Receptor for CXCL9, 10, 11), which is involved in trafficking of effector T cells, was significantly upregulated across multiple clusters (Fig. 3B) in GSD1b patients.

In addition, cluster 6, consisting of anti-inflammatory MΦ, exhibited decreased expression of CD163 (fold change GSD1b/control = 0.54, p-value = 0.002, Fig. 3A). CD163 is a cysteine-rich scavenger receptor on monocyte lineage cells involved in immune regulation and tissue homeostasis and has been shown to be strongly downregulated on MΦ and Monocytes in response to proinflammatory signalling[15], which may be consistent with the findings in the GSD1b patients, and may indicate impaired function of this anti-inflammatory macrophage subset. Cluster 27, activated emT_c_, showed higher expression of CXCR3 (fold change GSD1b/control = 1.78, p-value = 0.01, Fig. 3A), which is considered to represent an activation marker for CD8^+^ T cells; for this effector memory T cell population, it likely indicates activation and targeting to sites of infection[16]. The largest observed neutrophil population, cluster 18, CD15^high^-expressing cells, had an increase in MMI for CD45RO (fold change GSD1b/control = 1.763=, p-value = 0.02, Fig. 3A) and a decrease for CD45RA (fold change GSD1b/control = 0.735, p-value = 0.03, Fig. 3A). CD45RO is usually found on non-activated, resting neutrophils, while CD45RA is considered a marker of activation[17], which may suggest that this neutrophil population is less activated in GSD1b patients.

## Discussion

To date, the etiology of the immunodeficiency state and hyper-inflammatory intestinal condition observed in GSD1b remain unclear. Here, we utilize CyTOF technology to provide an in-depth overview of the innate and adaptive immune landscape in patients with GSD1b. Although our sample size was small, analysis of cell proportions in GSD1b patients showed little variability, indicating a robust assessment of significant changes not driven by variation within our groups. We were able to detect and assess 32 immune populations in both the innate and adaptive compartment, illuminating changes in GSD1b at a previously unprecedented depth.

Neutropenia is a key characteristic in patients harboring mutations in patients harboring deleterious mutations in *SLC37A4* leading to GSD1b. Although blood sample processing using lymphoprep gradient eliminates most circulating neutrophils, we still found small remaining neutrophil populations that showed upregulation of CD45RO and downregulation of CD45RA in patients with GSD1b, indicating an impairment in neutrophil function or lack of activation. A link to increased apoptosis in these neutrophils has been suggested in the past, along with a possible inability to switch to a glycolytic metabolism after activation in a hypoglycemic environment[18]. Furthermore, we show major changes in other immune cell populations. In line with the findings of Melis *et al*.[8] we observe a decrease in NK cells in GSD1b patients. The mechanism how GSD1b causes NK cell reduction is unclear, but might also be related to impaired glycolytic function in a hypoglycemic enviorment[19]. This is similar to the effect reported for lymphopenia and impairment of Warburg shift for T cells in GSD1b[8], although NK cells do not seem to undergo the switch to glycolytic energy generation as rapid as T cells[19]. As such, we observed a down-regulation in CD16 in NK cells in patients with GSD1b, likely due to the failure to sustain expansion and effector function in a hypoglycemic environment.

The strong reduction in anti-inflammatory MΦ (M2-like) is not explainable with the hypoglycemic environment being insufficient for sustaining a stable population after activation, because anti-inflammatory MΦ have been reported to rely mostly on oxidative phosphorylation (OXPHOS) to meet their needs while also maintaining some glycolytic activity[20], [21]. However, it is difficult to determine the association between the inflammatory state (M1-/M2-like) and effect of MΦ based on their surface marker expression (HLA-DR^high^ vs. HLA-DR^low^ in our case) alone. Some MΦ populations (e.g. M1-like) are known to undergo a switch to Warburg metabolism[20], [21]. Further characterization of MΦ populations, inflammatory status, and mode of activation in GSD1b patients might therefore be required.

We also observed a trend of increasing percentages of naïve T cells in GSD1b, consistent with the observation by Melis *et al*. of T cell dysregulation due to an impairment in glycolysis. However, we could not confirm their observation of a reduction in overall memory T cell levels. More striking is the observed transition from effector memory to central memory in T cells, a change that is predicted to result from the Warburg shift. Effector memory T cells, especially emT_c_ (cluster 27), were significantly reduced in GSD1b and, in contrast to cm T cells, are reported to rely heavily on glycolysis to meet their energy needs[22]-[24]. On the contrary, central memory T cells (cluster 11) were significantly increased in GSD1b and are reported to preferably perform oxidative phosphorylation, mainly fueled by fatty acid oxidation.[22]–[24] Our data shows a shift away from effector memory and towards the central memory T cell populations in GSD1b, which can be explained by the hypoglycemic conditions and impaired glycolysis in GSD1b favoring central memory over effector memory T cell phenotype.

We observed no significant changes in the B cell compartment similar to Melis *et al*.[8]. Naive B cells rely on OXPHOS with only a moderate increase of glycolysis upon activation[25], [26], explaining their ability to differentiate and form stable effector and memory populations, even in hypoglycemic conditions.

The reason for the global downregulation of myeloid markers in GSD1b remains unclear. The upregulation of CXCR3 in multiple clusters might indicate increased activation and targeting to inflamed tissue in GSD1b, especially the lung[27].

Although one explanation of the altered immune landscape is the hypoglycemia environment, this by itself cannot solely explain the immune phenotype observed in patients with GSD1b. An important comparison should be made to patients with GSD1a, resulting from *G6PC* mutations, that also suffer from severe recurrent hypoglycemia, but do not develop neutropenia or IBD. Therefore, it is likely that deleterious *SLC37A4* mutations impair immune function via additional mechanisms. Immunometabolomic Seahorse data on GSD1b T cells is sparse, but Melis *et al*. observed impaired glycolysis engagement in GSD1b patients[8]. Our results are in line with this conclusion, that glycolysis could explain the low levels of effector memory T cells and high levels of central memory T cells in GSD1b patients.

## Conclusion

In conclusion, the study presented here is to our knowledge the first to provide a detailed view of the immune landscape in GSD1b using a high-parameter panel. The alterations observed demonstrate that the underlying dysregulations of the immune system go much deeper than the commonly recognized neutropenia and affect multiple populations across all arms of the immune system, likely influencing the clinical manifestations of GSD1b such as recurrent infections and IBD. We speculate that the mechanism of the immune dysregulation in GSD1b is partially mediated by a failure of anaerobic glycolysis-dependent immune populations to differentiate and proliferate in the hypoglycemic environment commonly present in GSD1b patients, leading to immune deficiency associated with this rare disease. Further studies should address more specifically whether these immune defects are the result of metabolic defects, and whether specific interventions (e.g. SGLT2 inhibitors) ameliorate also these immune defects.

## Figures and Tables

**Table 1:** Subject Demographic and Clinical Data

Sample	M/F	Age	Condition	Drugs	Neupogen	CRP	ANC	Mutation
GSD1b 1	M	3.5	-	-	yes	1.34	460	NM_001164277.1 (SLC37A4): c.1042_1043delCT(p.Leu348Valfs*5)
GSD1b 2	M	11.5	Bronchiectasis	-	yes	0.55	780	
GSD1b 3	M	13.8	Bronchiectasis	-	yes	2.1	430	
GSD1b 4	M	7	IBD	Pentasa	yes	0.3	470	(SLC37A4):c.1211 delCT
GSD1b 5	M	12.5	IBD	Prednisone	yes	NA	900	(SLC37A4):c.1179G > A (p.Trp393*)
GSD1b 6	M	0.7	-	-	-	1.9	950	(SLC37A4):c.1041 delCT
control 1	F	14.5	control	-	-	NA	NA	control
control 2	F	17.5	control	-	-	NA	NA	control
control 3	M	17	control	-	-	NA	NA	control

Relevant clinical Information for GSD1b patients and controls. Age in years, CRP in mg/dl, ANC in cells per mm^3^. CRP and ANC determined at time of draw. Mutation annotation based on genetic testing performed at Sheba Medical Center.

## Data Availability

The datasets generated during and/or analysed during the current study are available from the corresponding author on reasonable request.
